# An Investigation into the Temporal Reproducibility of Tryptophan Metabolite Networks Among Healthy Adolescents

**DOI:** 10.1177/11786469211041376

**Published:** 2021-09-25

**Authors:** Kolade Oluwagbemigun, Andrea Anesi, Gerard Clarke, Matthias Schmid, Fulvio Mattivi, Ute Nöthlings

**Affiliations:** 1 Nutritional Epidemiology, Department of Nutrition and Food Sciences, University of Bonn, Germany; 2 Department of Food Quality and Nutrition, Research and Innovation Centre, Fondazione Edmund Mach, San Michele all’Adige, Italy; 3 APC Microbiome Ireland, University College Cork, Ireland; 4 INFANT Research Centre, University College Cork, Ireland; 5 Department of Psychiatry and Neurobehavioural Science, University College Cork, Ireland; 6 Department of Medical Biometry, Informatics and Epidemiology, University Hospital Bonn, University of Bonn, Germany; 7 Department of Cellular, Computational and Integrative Biology – CIBIO, University of Trento, San Michele all’Adige, Italy

**Keywords:** Tryptophan metabolite networks, temporal reproducibility, kynurenine, kynurenic acid, 3-hydroxykynurenine, 3-hydroxyanthranilic acid, xanthurenic acid, indole-3-acetic acid, Gaussian graphical model, Bayesian network

## Abstract

Tryptophan and its bioactive metabolites are associated with health conditions such as systemic inflammation, cardiometabolic diseases, and neurodegenerative disorders. There are dynamic interactions among metabolites of tryptophan. The interactions between metabolites, particularly those that are strong and temporally reproducible could be of pathophysiological relevance. Using a targeted metabolomics approach, the concentration levels of tryptophan and 18 of its metabolites across multiple pathways was quantified in 24-hours urine samples at 2 time-points, age 17 years (baseline) and 18 years (follow-up) from 132 (52% female) apparently healthy adolescent participants of the DOrtmund Nutritional and Anthropometric Longitudinally Designed (DONALD) Study. In sex-specific analyses, we applied 2 network approaches, the Gaussian graphical model and Bayesian network to (1) explore the network structure for both time-points, (2) retrieve strongly related metabolites, and (3) determine whether the strongly related metabolites were temporally reproducible. Independent of selected covariates, the 2 network approaches revealed 5 associations that were strong and temporally reproducible. These were novel relationships, between kynurenic acid and indole-3-acetic acid in females and between kynurenic acid and xanthurenic acid in males, as well as known relationships between kynurenine and 3-hydroxykynurenine, and between 3-hydroxykynurenine and 3-hydroxyanthranilic acid in females and between tryptophan and kynurenine in males. Overall, this epidemiological study using network-based approaches shed new light into tryptophan metabolism, particularly the interaction of host and microbial metabolites. The 5 observed relationships suggested the existence of a temporally stable pattern of tryptophan and 6 metabolites in healthy adolescent, which could be further investigated in search of fingerprints of specific physiological states. The metabolites in these relationships may represent a multi-biomarker panel that could be informative for health outcomes.

## Introduction

Tryptophan is an essential aromatic amino acid that is important for protein/peptide synthesis and turn-over.^1^ In the last years, tryptophan and its bioactive metabolites have attracted growing attention due to their association with health conditions such as systemic inflammation, cardiometabolic diseases, and neurodegenerative disorders.^1-8^ These metabolites arise from the 3 major host and microbial metabolic pathways of tryptophan catabolism: the oxidative or kynurenine pathway, the hydroxylation or serotonin pathway, and the microbial-derived indole pathway.^6,9-11^

There is cross-talk^12,13^ and a balanced interplay^14^ among these aforementioned pathways. Interestingly, the microbial production of indoles influences the host-derived pathways^15^ and provides a metabolic balance between the host-derived pathways.^11,16^ Therefore, the relationship of the full range of tryptophan metabolites may cut across multiple pathways, comprising intra- and inter-pathway host–host, microbial–microbial, and host–microbial metabolite interactions. Uncovering these broad and potentially novel interactions among the metabolites of tryptophan is imperative. Moreover, little is known whether steps in the tryptophan metabolic pathways are recoverable exclusively from the concentration of its individual catabolites within biological samples. Indeed, the observed relationships between tryptophan metabolites would potentially carry a greater deal of health-related information than its single metabolites.

Furthermore, strongly related biomarkers are associated with unique biological functions^17,18^ and the relationship between biomarkers have been shown to change during disease development and progression.^19^ Given the intricate interplay of the physiological functions of tryptophan metabolites,^2,20-22^ the relationships between tryptophan metabolites that are strong and reproducible over time could represent fingerprints of pathophysiological states. In fact, a multi-biomarker panel of the tryptophan metabolites in these relationships may be highly predictive of health outcomes.

Network analysis offers a way to explicitly explore the dynamic interactions among metabolites.^22^ It allows the identification of known and non-intuitive metabolite relationships and brings the interactions between different pathways and physiological functions to the fore.^22^ Thus, this analytical approach presents an alternative strategy to understanding the relationship among tryptophan metabolites. The 2 commonly used network analysis approaches are the Gaussian graphical model (GGM) and Bayesian network (BN).^23^ Considering that spurious results can arise in purely data-driven network analysis,^24^ converging findings from 2 or more network approaches will reduce statistical artifacts and ensure the identification of reliable relationships between the metabolites of tryptophan. In addition, tryptophan metabolism is sex-dependent^1^ and sex hormones interact with some tryptophan pathways,^25,26^ thus the relationships among tryptophan metabolites may be different between sexes.

Considering that no study until date has reported on the temporal reproducibility of the relationship among metabolites of tryptophan catabolism, the present study sought to explore sex-specific tryptophan metabolite networks using GGM and BN, retrieve the strongly related tryptophan metabolites, and determine whether their relationship is temporally reproducible.

## Subject and Methods

### Study design and population

The current study was conducted within the framework of the DOrtmund Nutritional and Anthropometric Longitudinally Designed (DONALD) Study. The DONALD Study is an ongoing, open population-based cohort study designed to address complex research questions on diet, growth, development, and metabolism from infancy to adulthood. The DONALD Study has been described in detail elsewhere.^27^ In brief, participants were recruited in the city of Dortmund and surrounding communities through personal contacts, maternity wards, or pediatric practices. Eligible participants were healthy (with no prevalent diseases) German infants whose parents were willing to participate in a long-term study and had sufficient knowledge of the German language. Due to its specific design, individuals from well-educated mothers were overrepresented in this study. The Ethics Committee of the University of Bonn (approval number 098/06) approved the study and written informed consent was obtained from all participants. The study was conducted according to the guidelines of the Declaration of Helsinki. The annual examinations in the DONALD Study comprise 3-day weighed dietary records, anthropometric measurements, and collection of 24-hours urine samples.

For this present study, we considered a subset of participants from which the concentration levels of a set of metabolites were repeatedly profiled by a targeted metabolomic approach in 24-hours urine samples.^8^ These metabolites included tryptophan and 18 of its metabolites. We considered 132 individuals from whom these metabolites were profiled at 2-time points, ages 17 and 18 years. Thus, age 17 and 18 years were considered as the baseline and follow-up time-points, respectively.

### Urine sampling, preparation, and targeted metabolomic profiling

#### Urine sampling

The participants collected their 24-hours urine at home following standardized procedure and detailed instruction. They were asked to void their bladders upon getting up in the morning. This first sample was discarded. Afterwards, the collection began. The collection ended with collecting first morning urine on the next day. The samples were stored in preservative-free, Extran-cleaned (Extran, MA03; Merck, Darmstadt, Germany) 1 L plastic containers at less than −12°C. Finally, samples were transferred to the study centre where they were stored at −22°C until metabolomic analysis. The 24-hours urine sampling in the DONALD Study has been presented in details elsewhere.^28^

#### Urine sample preparation and targeted metabolomic profiling

**Figure 1.**
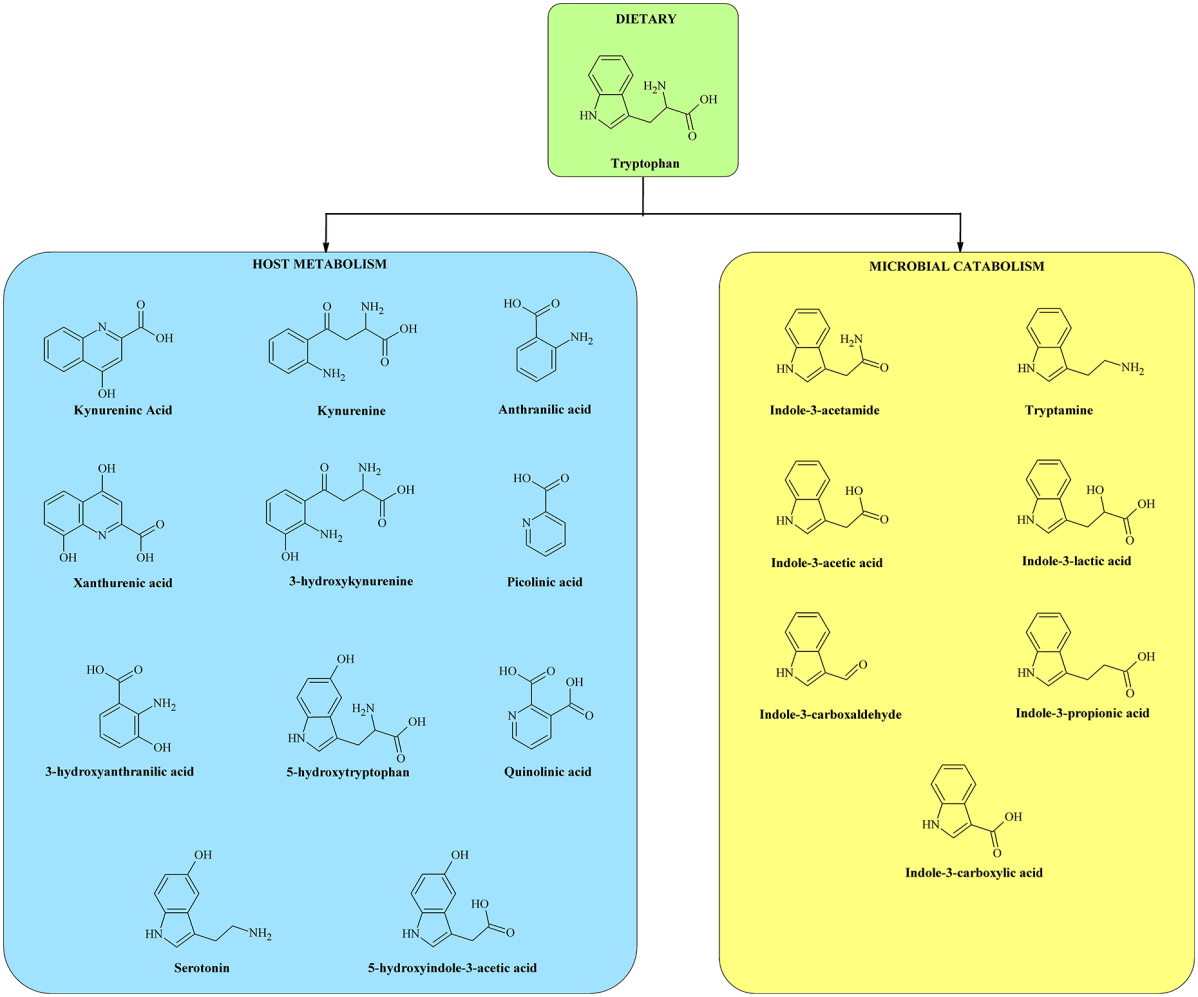
The structures of tryptophan and 18 of its metabolites as products of host and microbial metabolism.

For each of the 24-hours urine sample, 25 µL aliquot and 225 µL of internal standard mix with water: acetonitrile 8:2 (v/v) were added (trypthophan-d5 at final concentration 1 ppm; tyrosine-d4, methionine-d4, serotonin-d4, kynurenic acid-d5, 5-hydroxyindole-acetic acid-d5, and dopamine-d5 at 0.5 ppm), were loaded in 96 well multifilter plates (Millipore) and were filtered by applying positive pressure (nitrogen) at 3 PSI. Ultra-high-performance liquid chromatography coupled with electrospray ionization triple quadrupole mass spectrometry, UHPLC-ESI-QqQ-MS (Xevo TQ-MS, Waters^®^) was employed to determine the concentration of tryptophan and 18 of its downstream metabolites in 2 µL urine. Data processing was performed with Mass Lynx 4.1 software (Waters^®^). In addition to tryptophan, the metabolites quantified were picolinic acid, quinolinic acid, kynurenic acid, kynurenine, xanthurenic acid, anthranilic acid, 3-hydroxyanthranilic acid, 3-hydroxykynurenine, serotonin, 5-hydroxyindole-3-acetic acid, 5-hydroxy-tryptophan, indole-3-acetamide, indole-3-acetic acid, indole-3-lactic acid, indole-3-propionic acid, indole-3-carboxaldehyde, indole-3-carboxylic acid, and tryptamine. Our previous publication provides more details of the experimental procedures for their measurement.^29^ Figure 1 shows the structures of the 18 metabolites as products of host and microbial metabolism.

### Assessment of covariates

Sex and birth size, birth weight (g) and length (cm) were retrieved from maternal gestational records. Dietary intake was assessed by using 3-day weighed dietary records. From these dietary records, we calculated individual means of energy and protein intake for baseline and follow-up, using our continuously updated in-house food composition database. Experienced nurses conducted anthropometric measurements, which include measurements of body weight and height.^27^ Body mass index (BMI) was calculated as weight (kg)/height (m)^2^ at baseline and follow-up.

### Statistical analyses

#### Study sample characteristics

All participants had complete data on all metabolites and covariates. The basic characteristics of the study population by sex were presented as medians with 25th and 75th percentiles. Sex differences in baseline characteristics were estimated using the Mann–Whitney *U* test. The analyses were performed with SAS 9.4 (SAS Institute, Cary, NC).

#### Tryptophan metabolite networks

We explored sex-specific tryptophan metabolite networks using 2 methods. The first method, the GGM (or partial correlation network) is an exploratory tool that simultaneously estimates and selects the partial correlations between variables.^23,30^ Thus, the correlation between 2 variables in the GGM is independent of all other variables in the network. The second method, the BN is a graphical model that encodes probabilistic relationships among variables of interest.^31,32^ In the biological context, BNs can be used to understand the network of dependencies among the variables, to pinpoint the strongest dependencies and clear independencies.^33^ Thus, the strength of the association between 2 variables in GGM and BN is the partial correlation coefficient and probability, respectively. The current analyses used the baseline (age 17 years) and the follow-up (age 18 years) concentration levels of tryptophan and 18 of its metabolites in 24-hours urine to develop GGM and BN. GGM and BN were constructed without any a priori assumptions on the relationships between the metabolites. To reduce the influence of outliers, we standardized all variables including covariates to a mean of 0 and standard deviation of 1 before entering them into the analysis.

In each sex, we constructed GGM using a regularization technique, the graphical least absolute shrinkage and selection operator (gLASSO).^34^ The gLASSO, a fast variant of the LASSO, helps control spurious correlations and only requires a covariance matrix. A range of networks was estimated under different values of tuning parameter, λ and the network that maximizes the Bayesian information criterion (BIC) was selected as the optimal network. We used the BIC because it has been shown to provide a good balance of high sensitivity and high specificity of connections.^35^ We implemented the graphical LASSO algorithm using the *bootnet* package in R-4.0.3. Starting with the baseline GGM; we considered metabolites that were strongly related, that is, pairs of metabolites with regularized partial correlations ⩾.3. This cutoff threshold of 0.3 was used in another metabolite network analysis.^36^ To confirm the sex-specific analysis, we compared the overall network structure of males and females visually (graphically) and by using a permutation-based network structure invariance test^37^ in R-4.0.3. This invariance test estimates whether the networks of the sexes differed in their relationships among metabolites. This test is valid when sample sizes are comparable, as in our case. Afterwards, we adjusted the baseline GGM for selected non-metabolite covariates, specifically birth weight and length, BMI, and energy and protein intake. Finally, we constructed the covariate-adjusted GGM for the follow-up. We extracted temporally reproducible associations as those strongly related metabolites observed in both the adjusted baseline and follow-up GGMs.

Secondly, we applied the BN to the same datasets. Just like GGM, we constructed sex-specific baseline BN, covariate-adjusted baseline BN, and covariate-adjusted follow-up BN using the BIC hill-climbing algorithm with the *bnlearn* package in R-4.0.3.^38^ We used the BIC as the scoring metric because it has been shown to outperform other scoring functions in recovering underlying structures.^39^ For each dataset, we generated network structures from 1000 bootstrap samples and the final network was the aggregate of all bootstrap samples. The strength of the metabolite associations was quantified as the relative frequency of connections from the 1000 bootstrap samples. We considered the commonly used default relative frequency of 0.85 for strongly related metabolites. This corresponds to the connections that appear in at least 85% of the 1000 bootstrap samples. To test the difference between the baseline BNs of the sexes, we computed the Hamming distance^40^ that represents the number of associations that differ between male and females. For this analysis, we presented BN as undirected networks and interpreted only the strength of the probabilistic relationships because our goal was to represent dependency among metabolites that is not necessarily causal. Besides, our sample size might be insufficient to draw reliable directionality conclusions and the orientations of connections in BN of observational data is sometimes counterintuitive.^41^

We considered reliable and valid relationships as the strongly related and temporally reproducible connections shared by both GGM and BN.

## Results

### Study population characteristics

The study population (n = 132) comprised 64 males and 68 females. Table 1 describes the basic characteristics of the study population. At *P* ⩽ .05, the birth weight and length were higher in males than in females. At both baseline and follow-up, energy and protein intake as well as the 24-hours urine concentration levels of tryptophan, kynurenine, kynurenic acid, and tryptamine levels were higher in males than in females. Conversely, at both time-points, the concentration levels of indole-3-lactic acid and indole-3-carboxaldehyde were higher in females than in males.

**Table 1.** Basic characteristics of the study population.

	All (n = 132)	Male (n = 64)	Female (n = 68)	*P* value				
Birth weight, g	3500 (3145, 3800)	3665 (3275, 3900)	3365 (3100, 3655)	<.001				
Birth length, cm	51.5 (50, 53)	52 (51, 54)	51 (50, 52.5)	.01				
	All (n = 132)	Male (n = 64)	Female ( = 68)	*P* value	All (n = 132)	Male (n = 64)	Female (n = 68)	*P* value
	Baseline				Follow-up			
Energy, kcal/day	2131.75 (1785.46, 2664.09)	2622.9 (2296.47, 2803.29)	1821.59 (1627.71, 2018.56)	<.001	2186.77 (1883.17, 2724.57)	2713.01 (2263.52, 3018.51)	1924.11 (1686.05, 2095.29)	<.001
Protein, g/day	73.87 (58.65, 90.27)	90.32 (79.75, 101.4)	61.06 (51.99, 68.43)	<.001	75.36 (59.79, 93.02)	92.15 (78.26, 103.64)	64.87 (51.92, 72.43)	<.001
Body mass index, kg/m²	21.34 (19.77, 23.19)	21.56 (19.99, 23.54)	20.87 (19.53, 22.95)	.24	21.76 (20.28, 23.35)	22.05 (20.61, 23.39)	21.55 (19.93, 22.97)	.16
Metabolites, µM								
Tryptophan	55.29 (37.23, 92.33)	71.31 (38.35, 100.42)	47.71 (36, 84.54)	.03	53.65 (39.94, 82.47)	67.81 (43.81, 91.88)	48.84 (29.47, 68.53)	.01
Picolinic acid	1.43 (1.23, 1.59)	1.42 (1.22, 1.6)	1.43 (1.24, 1.55)	.71	1.46 (1.29, 1.63)	1.46 (1.35, 1.64)	1.47 (1.23, 1.62)	.56
Quinolinic acid	40.08 (27.44, 49.58)	40.55 (28.34, 48.37)	39.1 (25.53, 52.99)	.65	35.49 (28.18, 46.65)	35.07 (27.01, 48.02)	36.51 (29.49, 46.06)	.79
Kynurenic acid	20.1 (13.96, 28.73)	22.18 (15.26, 31.04)	17.57 (11.04, 26.53)	.01	17.37 (11.51, 29.14)	21.14 (12.98, 34.57)	15.16 (9.26, 23.69)	.002
Kynurenine	3.55 (1.92, 6.13)	4.23 (2.35, 7.78)	2.46 (1.65, 5.26)	.001	3.19 (1.93, 5.25)	3.88 (2.47, 6.19)	2.53 (1.65, 4.18)	.003
Xanthurenic acid	6.38 (3.92, 9.04)	5.77 (4.2, 7.87)	6.68 (3.82, 9.75)	.56	5.83 (3.75, 9.48)	5 (3.75, 9.3)	6.62 (3.74, 10.07)	.60
Anthranilic acid	0.4 (0.27, 0.59)	0.39 (0.26, 0.56)	0.42 (0.28, 0.62)	.38	0.37 (0.25, 0.52)	0.37 (0.25, 0.48)	0.37 (0.25, 0.55)	.89
3-hydroxyanthranilic acid	0.43 (0.25, 0.71)	0.42 (0.25, 0.69)	0.43 (0.24, 0.72)	.64	0.4 (0.23, 0.74)	0.38 (0.24, 0.69)	0.41 (0.21, 0.82)	.63
3-hydroxykynurenine	0.34 (0, 0.59)	0.33 (0, 0.55)	0.36 (0.06, 0.63)	.58	0.29 (0, 0.52)	0.33 (0, 0.59)	0.27 (0, 0.5)	.50
Serotonin	0.45 (0.32, 0.66)	0.45 (0.47, 0.62)	0.45 (0.3, 0.64)	.34	0.47 (0.29, 0.64)	0.43 (0.3, 0.67)	0.48 (0.28, 0.58)	.48
5-hydroxyindole-3-acetic acid	18.47 (13.85, 29.56)	18.61 (14.09, 29.48)	18.35 (12.89, 30.19)	.93	18.98 (12.4, 27.34)	19.99 (12.49, 27.11)	18.09 (12.29, 28.75)	.97
	All (n = 132)	Male (n = 64)	Female ( = 68)	*P* value	All (n = 132)	Male (n = 64)	Female (n = 68)	*P* value
	Baseline				Follow-up			
Indole-3-acetamide	0.26 (0.18, 0.43)	0.29 (0.19, 0.43)	0.25 (0.15, 0.39)	.22	0.26 (0.17, 0.41)	0.32 (0.16, 0.47)	0.23 (0.17, 0.36)	.25
Indole-3-acetic acid	30.72 (19.4, 42.67)	30.36 (22.4, 42.67)	31.78 (17.56, 42.21)	.59	27.34 (19.13, 41.67)	27.56 (18.19, 42.18)	27.1 (21.63, 41.44)	.84
Indole-3-lactic acid	1.29 (0.72, 2.02)	0.98 (0.69, 1.62)	1.65 (0.85, 2.7)	<.001	1.25 (0.83, 1.93)	0.99 (0.71, 1.44)	1.5 (1.08, 2.82)	<.001
Indole-3-propionic acid	0.05 (0.04, 0.07)	0.05 (0.04, 0.07)	0.05 (0.04, 0.07)	.36	0.05 (0.04, 0.08)	0.05 (0.04, 0.08)	0.06 (0.04, 0.08)	.12
Indole-3-carboxaldehyde	0.13 (0.08, 0.19)	0.12 (0.08, 0.16)	0.14 (0.08, 0.26)	.04	0.12 (0.07, 0.2)	0.11 (0.07, 0.15)	0.13 (0.08, 0.27)	.04
Indole-3-carboxylic acid	0.09 (0.07, 0.13)	0.1 (0.07, 0.13)	0.09 (0.06, 0.15)	.44	0.09 (0.7, 0.13)	0.1 (0.07, 0.13)	0.09 (0.07, 0.13)	.83
Tryptamine	0.42 (0.29, 0.61)	0.45 (0.34, 0.76)	0.41 (0.26, 0.57)	.02	0.41 (0.25, 0.63)	0.47 (0.32, 0.67)	0.36 (0.22, 0.56)	.03

All values are in median with 25 and 75 percentile, n, count.

### Tryptophan metabolite networks

**Figure 2.**
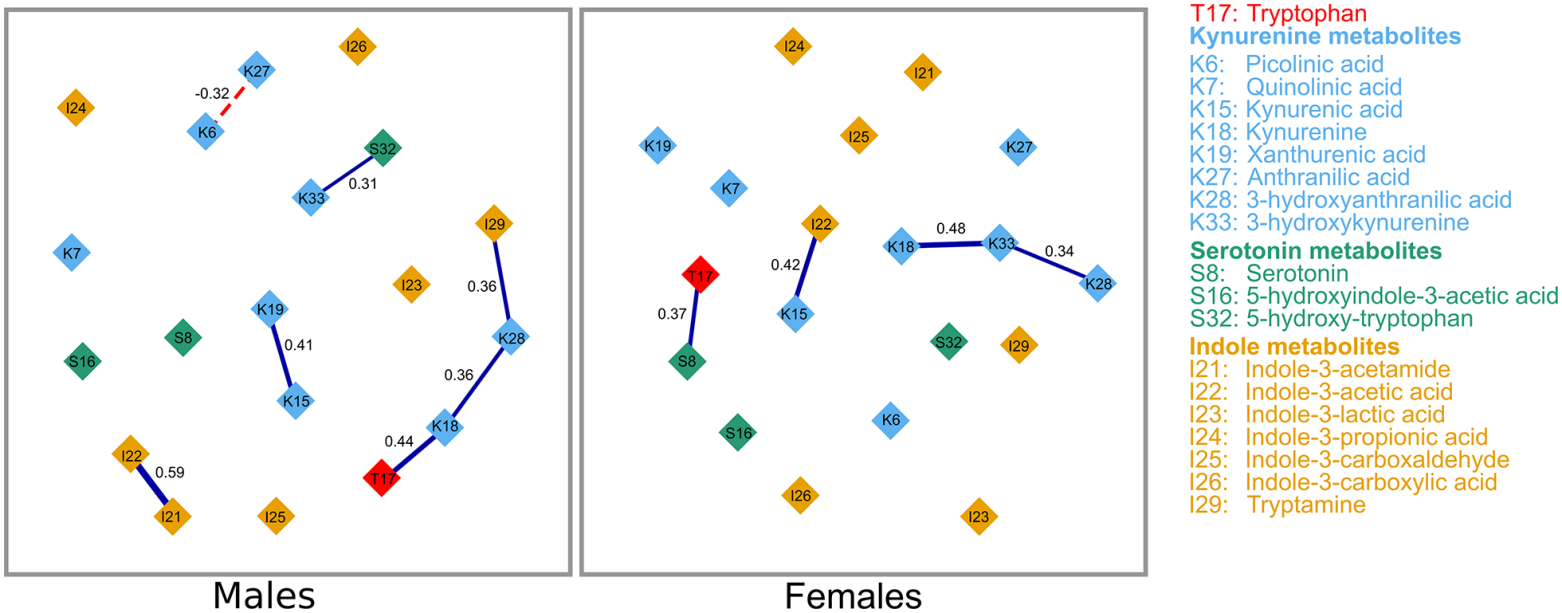
Gaussian graphical model of tryptophan and its metabolites at baseline. Only strong correlations, with regularized correlation coefficients ⩾.3, are shown. Connections between metabolites represent partial regularized correlation coefficients. Positive and negative connections are shown in blue bold and red dotted lines, respectively. The correlation coefficients are displayed and are depicted by the thickness of the connections.

Out of the 171 possible pairwise correlations in the baseline GGM of tryptophan and 18 of its metabolites, males had a slightly denser network (82 non-zero correlations) than females (76 non-zero correlations). There also appear to be a higher proportion of negative correlations in males than in females and some connections that are present in 1 sex were absent in the other. The network comparison test showed that the overall structure of the baseline GGM of the sexes with comparable sample size (males, n = 64 and females, n = 68) are not identical (*P* = .002). This indicates that the GGMs of the sexes featured different connections. As displayed in Figure 2, out of the non-zero correlations, 7 (8.5%) in males and 4 (5.5%) in females were strongly related (regularized partial correlations, *r* ⩾ .3). All the strongly related metabolites in females were positive; however, all but 1 was positive in males. The strongest correlations were between indole-3-acetamide and indole-3-acetic acid in males, and between kynurenine and 3-hydroxykynurenine in females.

The internal accuracy analyses of the baseline GGM, using the connection difference test supported that the strongly related metabolites were significantly different from most of the other non-zero correlations in the network (Supplemental Figure S1A and B). This indicates that these associations are internally reliable.

**Figure 3.**
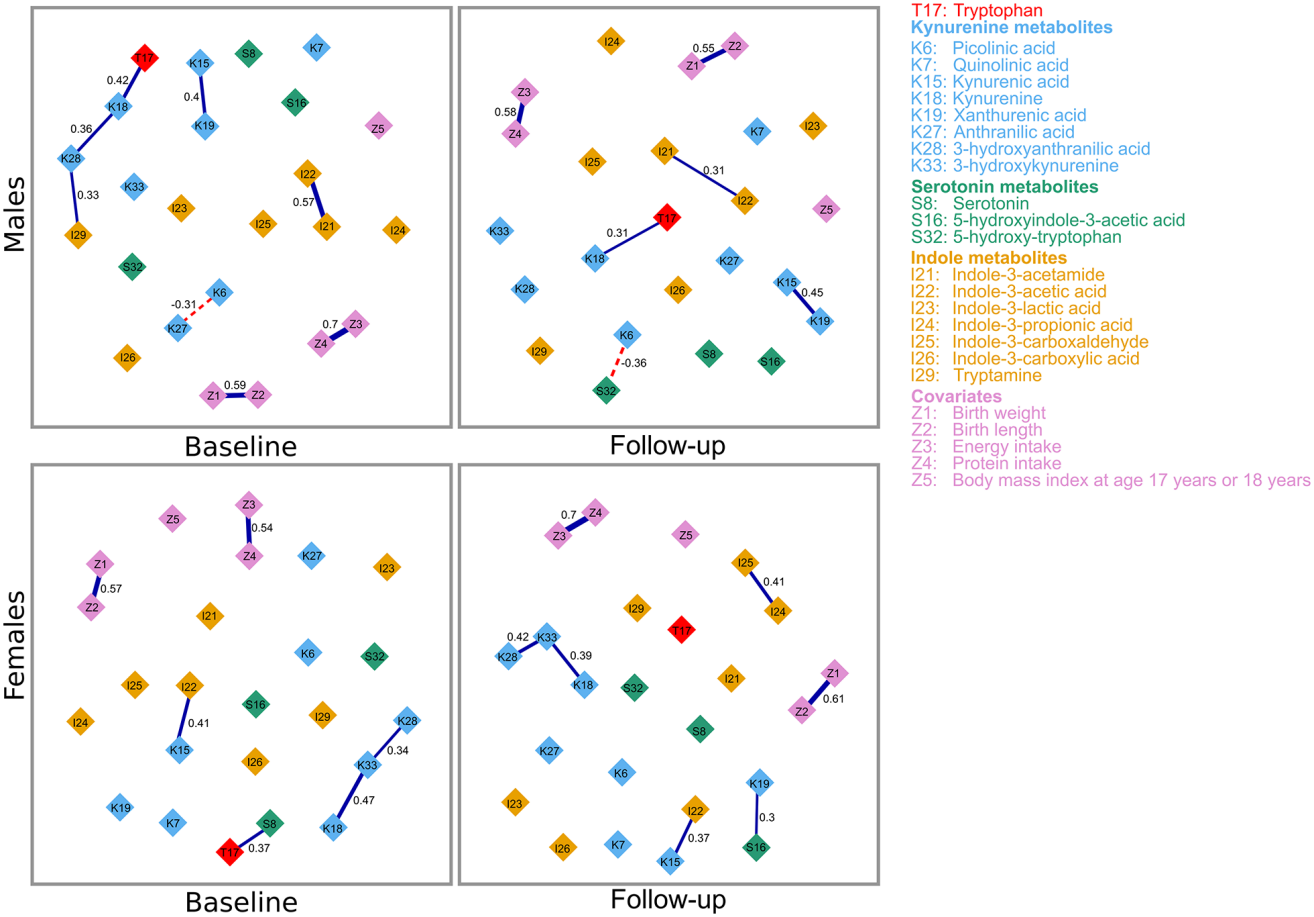
Covariate-adjusted Gaussian graphical model of tryptophan and its metabolites in both sexes at baseline and follow-up. Connections between metabolites represent partial regularized correlation coefficients. Positive and negative connections are shown in blue bold and red dotted lines, respectively. The correlation coefficients are displayed and are depicted by the thickness of the connections.

After adjusting for the covariates, birth weight and length, BMI, and energy and protein intake, the strongly related metabolites with their regularized partial correlations in the baseline and follow-up GGM of both sexes are shown in Figure 3. In the covariate-adjusted baseline GGM in males (Figure 3, upper panel, left), the strongly related metabolites were indole-3-acetamide–indole-3-acetic acid (*r* = .57), tryptophan–kynurenine (*r* = .42), kynurenic acid–xanthurenic acid (*r* = .4), kynurenine–3-hydroxyanthranilic acid (*r* = .36), 3-hydroxyanthranilic acid–tryptamine (*r* = .33), and picolinic acid–anthranilic acid (*r* = −.31). At follow-up (Figure 3, upper panel, right), kynurenic acid–xanthurenic acid (*r* = .45), picolinic acid–5-hydroxy-tryptophan (*r* = −.36), indole-3-acetamide–indole-3-acetic acid (*r* = .31), and tryptophan–kynurenine (*r* = .31) were strongly related. Three temporally reproducible associations, indole-3-acetamide–indole-3-acetic acid, tryptophan–kynurenine, and kynurenic acid–xanthurenic acid emerged. At follow-up, the partial correlations of indole-3-acetamide–indole-3-acetic acid and tryptophan–kynurenine were negatively attenuated, while kynurenic acid–xanthurenic acid was positively attenuated.

In the covariate-adjusted baseline GGM in females (Figure 3, lower panel, left), the strongly related metabolites were kynurenine–3-hydroxykynurenine (*r* = .47), kynurenic acid–indole-3-acetic acid (*r* = .41), tryptophan–serotonin (*r* = .37), and 3-hydroxykynurenine–3-hydroxyanthranilic acid (*r* = .34), and at follow-up (Figure 3, lower panel, right), kynurenic acid–indole-3-acetic acid (*r* = .45); 3-hydroxykynurenine–3-hydroxyanthranilic acid (*r* = .42), kynurenine–3-hydroxykynurenine (*r* = .39), indole-3-propionic acid–indole-3-carboxaldehyde (*r* = .31), and xanthurenic acid–5-hydroxyindole-3-acetic acid (*r* = .3). The 3 temporally reproducible associations were kynurenic acid–indole-3-acetic acid, kynurenine–3-hydroxykynurenine, and 3-hydroxykynurenine–3-hydroxyanthranilic acid. The kynurenic acid–indole-3-acetic acid and 3-hydroxykynurenine–3-hydroxyanthranilic acid were positively attenuated, while kynurenine–3-hydroxykynurenine was negatively attenuated.

**Figure 4.**
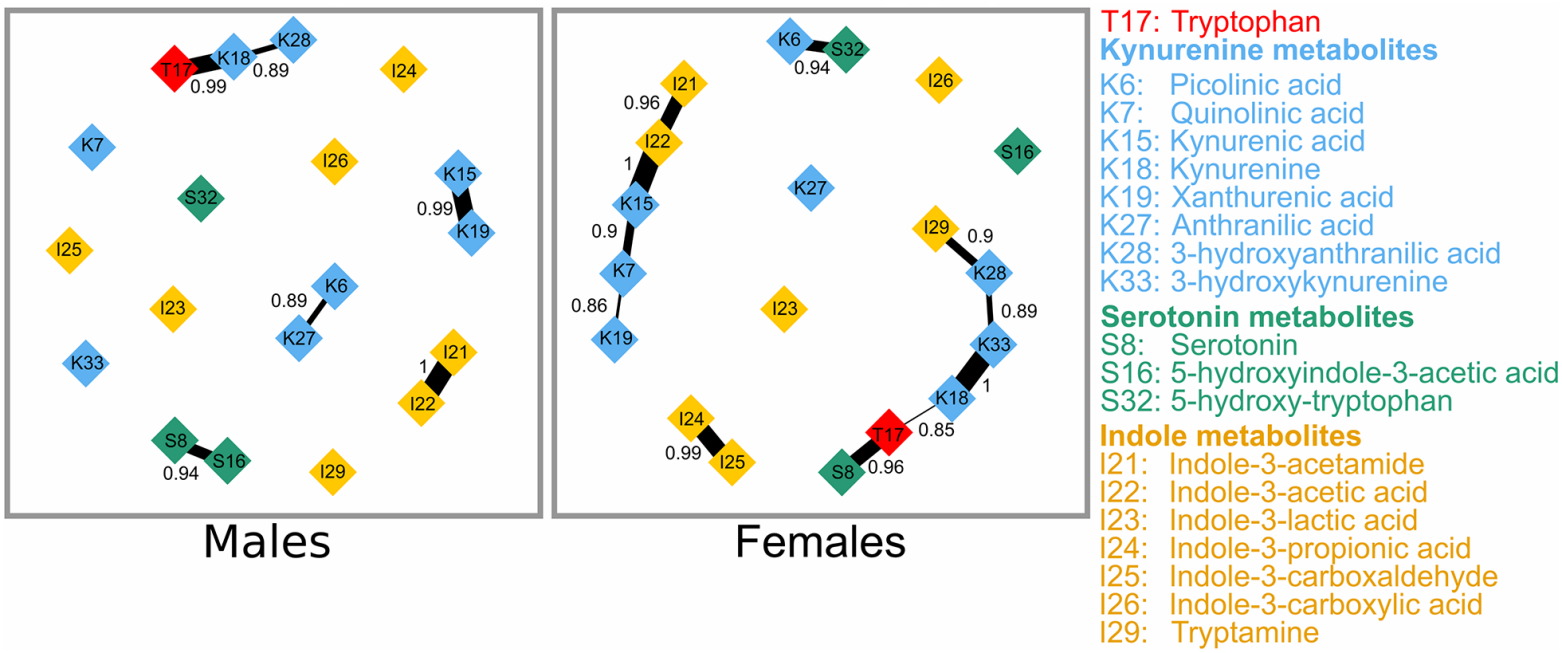
Bayesian network of tryptophan and its metabolites at baseline. Connections between metabolites represent probability strength. The probability strengths are displayed and are depicted by the thickness of the connections.

The result of the baseline BN of tryptophan and 18 of its metabolites for males and females is presented in Figure 4. The Hamming distance showed that 13 associations differ between the sexes. There were 6 and 11 highly probable associations between metabolites in males and females, respectively.

**Figure 5.**
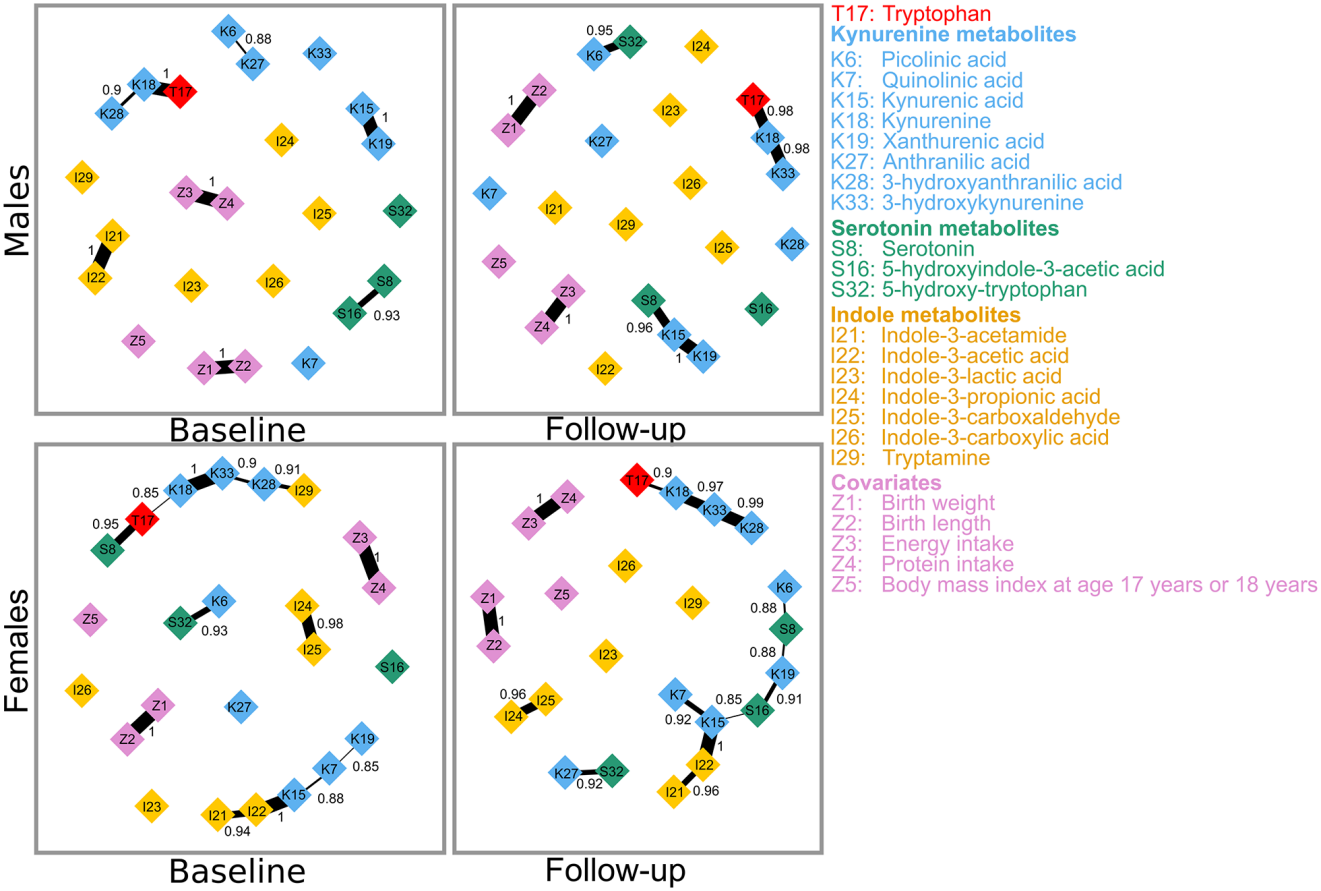
Covariate-adjusted Bayesian network of tryptophan and its metabolites. Connections between metabolites represent probability strength. The probability strengths are displayed and are depicted by the thickness of the connections.

The covariate-adjusted baseline BN in males (Figure 5, upper panel, left) showed that the highly probable associations were indole-3-acetamide–indole-3-acetic acid (strength = 1), tryptophan–kynurenine (strength = 1), kynurenic acid–xanthurenic acid (strength = 1), serotonin–5-hydroxyindole-3-acetic acid (strength = 0.93), kynurenine–3-hydroxyanthranilic acid (strength = 0.9), picolinic acid–anthranilic acid (strength = 0.88). At follow-up (Figure 5, upper panel, right), 4 associations, indole-3-acetamide–indole-3-acetic acid, serotonin—5-hydroxyindole-3-acetic acid, kynurenine–3-hydroxyanthranilic acid, and picolinic acid–anthranilic acid were absent and 3 new ones, picolinic acid–5-hydroxy-tryptophan, kynurenic acid–serotonin, and kynurenine–3-hydroxykynurenine emerged. The 2 temporally reproducible associations in males were tryptophan–kynurenine and kynurenic acid–xanthurenic acid.

In the covariate-adjusted BN in females (Figure 5, lower panel, left), we observed that kynurenic acid–indole-3-acetic acid (strength = 1), kynurenine–3-hydroxykynurenine (strength = 1), indole-3-propionic acid–indole-3-carboxaldehyde (strength = 0.98), tryptophan–serotonin (strength = 0.95), indole-3-acetamide–indole-3-acetic acid (strength = 0.94), picolinic acid–5-hydroxy-tryptophan (strength = 0.93), 3-hydroxyanthranilic acid–tryptamine (strength = 0.91), 3-hydroxykynurenine–3-hydroxyanthranilic acid (strength = 0.90), kynurenic acid–quinolinic acid (strength = 0.88), tryptophan–kynurenine (strength = 0.85), and quinolinic acid–xanthurenic acid (strength = 0.85) were highly related. At follow-up (Figure 5, lower panel, right), 4 associations, tryptophan–serotonin, picolinic acid–5-hydroxy-tryptophan, 3-hydroxyanthranilic acid–tryptamine, quinolinic acid–xanthurenic acid were absent and 5 new associations, picolinic acid–serotonin, kynurenic acid–5-hydroxyindole-3-acetic acid, xanthurenic acid–serotonin, xanthurenic acid–5-hydroxyindole-3-acetic acid, and anthranilic acid–5-hydroxy-tryptophan, emerged. The 7 temporally reproducible associations in females were indole-3-acetamide–indole-3-acetic acid, indole-3-propionic acid–indole-3-carboxaldehyde, kynurenic acid–indole-3-acetic acid, kynurenic acid–quinolinic acid, kynurenine–3-hydroxykynurenine, 3-hydroxykynurenine–3-hydroxyanthranilic acid, and tryptophan–kynurenine.

**Figure 6.**
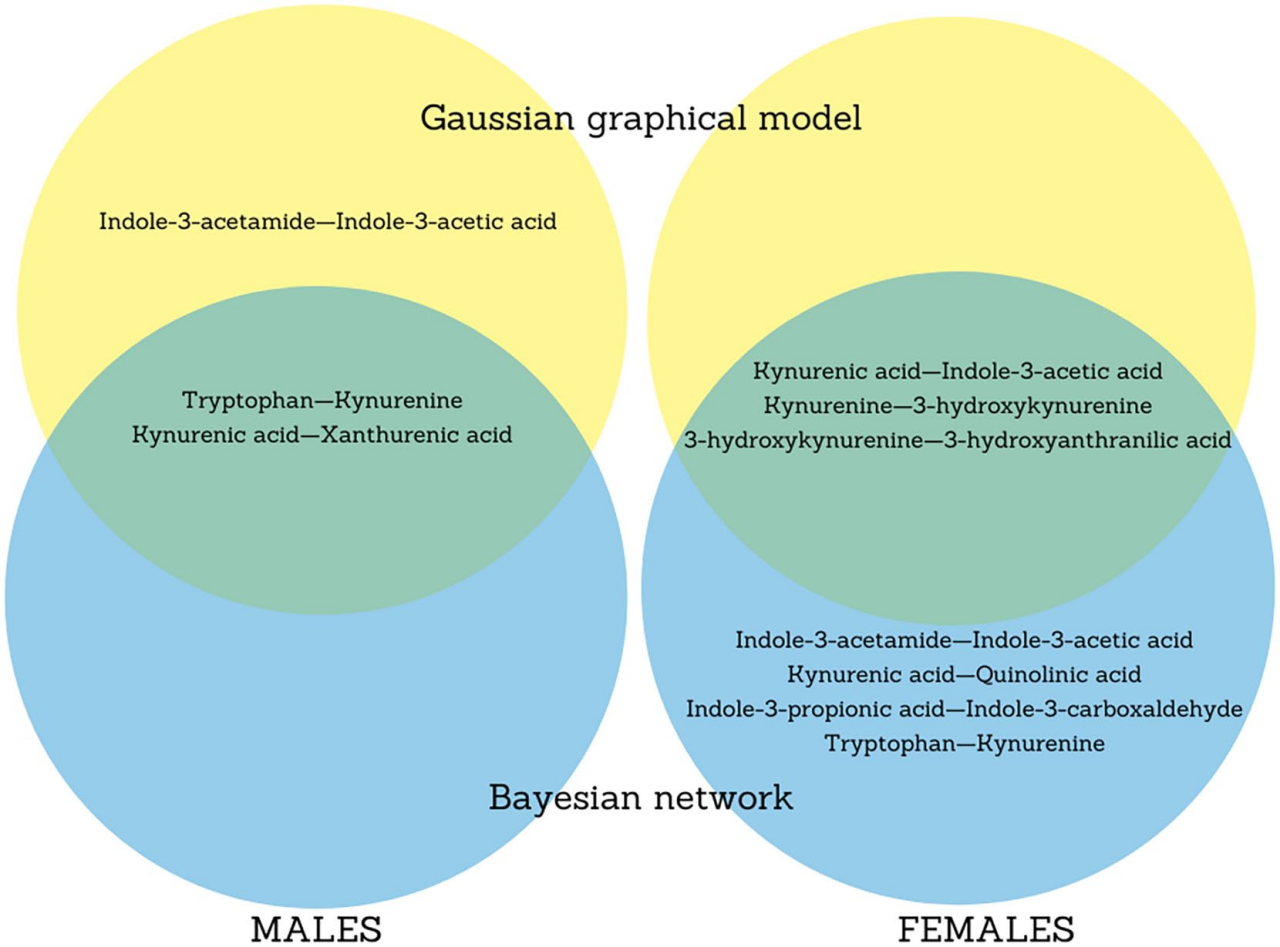
Venn diagram showing the strong and temporally reproducible relationships between tryptophan and its metabolites that overlap in 2 network approaches.

Figure 6 summarizes the strong temporally reproducible associations that are shared in both GGM and BN for each sex. Overall, there were 2 associations, between tryptophan and kynurenine, and between kynurenic acid and xanthurenic acid in males, as well as 3 associations, between kynurenine and 3-hydroxykynurenine, between 3-hydroxykynurenine and 3-hydroxyanthranilic acid, and between kynurenic acid and indole-3-acetic acid in females. These associations comprised 7 metabolites, namely tryptophan, kynurenine, kynurenic acid, 3-hydroxykynurenine, 3-hydroxyanthranilic acid, xanthurenic acid, and indole-3-acetic acid.

## Discussion

The current epidemiological study explored tryptophan metabolite networks in the 24-hours urine of 132 adolescents using 2 approaches, GGM and BN, with a specific focus on the temporal reproducibility of the strongly related metabolites shared by the 2 approaches. Two notable findings emerged: (1) we identified 5 associations that are strong and temporally reproducible and (2) these associations were distinct for males and females. The 5 associations were the novel relationships between kynurenic acid and indole-3-acetic acid in females and between kynurenic acid and xanthurenic acid in males as well as the well-known relationships between kynurenine and 3-hydroxykynurenine, and between 3-hydroxykynurenine and 3-hydroxyanthranilic acid in females and between tryptophan and kynurenine in males. The 5 observed relationships, which were host–microbial and intra-pathway host–host metabolite interactions, suggested the existence of a stable pattern of tryptophan and 6 metabolites in healthy adolescent, which could be further investigated in search of fingerprints of specific physiological states. Furthermore, the 7 metabolites in these relationships—the precursor tryptophan, the host-derived metabolites, kynurenine, kynurenic acid, 3-hydroxykynurenine, 3-hydroxyanthranilic acid, xanthurenic acid, and the gut-microbiota derived indole-3-acetic acid—could be considered a multi-biomarker panel of metabolites that have reproducible patterns in the urine of healthy adolescents. As such, any perturbation of these multiple correlations may be informative for health outcomes.

The first novel relationship was between the host-derived kynurenic acid and the microbial-derived indole-3 acetic acid. This interesting finding indicates that the relationship between tryptophan metabolites goes across host and microbial pathways. Except for the temporal stability of kynurenic acid,^42,43^ neither the temporal stability of indole-3-acetic acid nor the association between kynurenic acid and indole-3-acetic acid have been previously reported. Similar to the well-known gut microbiome-mediated tryptophan catabolism that produces indole-3-acetic acid,^44^ prevailing evidence suggests the gut microbial-mediated catabolism of kynurenic acid and its conversion into quinaldic acid.^45^ In fact, there is coevolutionary commensalism between host and microbes in the catabolism of tryptophan.^46^ Until now, there is no evidence that any of these metabolites is a substrate or a modulator of the enzymes involved in the production of the other. Clearly, kynurenine is the primary source of kynurenic acid.^6,20^ However, there is evidence that some kynurenic acid is also formed from an indole metabolite, indole-3-pyruvic acid,^47^ and indole-3-pyruvic acid pathway is one of the bacterial indole-3-acetic acid biosynthetic pathways,^48^ hence it is possible that indole-3-acetic acid has downstream effects on kynurenic acid or the host metabolic pathways in general. Therefore, this strong and temporally reproducible association between kynurenic acid and indole-3 acetic acid may represent one of the direct metabolite exchanges along the host-microbe metabolic axes that are crucial in the interaction between host and the gut microbiome. Further, these 2 metabolites are ligands for the Aryl Hydrocarbon Receptor (AHR), a ligand-activated transcription factor with a role in xenobiotic metabolism, immune regulation, and mucosal barrier function,^49,50^ thus their relationship might reflect their joint activation of this receptor. Additionally, the association between them might indicate a concomitant reduction of their catabolic pathways.

Another novel finding is the relationship between 2 host-derived metabolites, kynurenic acid and xanthurenic acid. A direct biochemical link between these 2 kynurenine pathway metabolites have not been specifically reported before. Nonetheless, this finding lends credence to reports that some aspects of tryptophan metabolism are still largely unknown^51^ and that strongly related metabolites do not always correspond to known biochemical connections.^17^ Clearly, the temporally reproducible association between kynurenic acid and xanthurenic acid in our study is consistent with the temporal reproducibility of each of kynurenic acid and xanthurenic acid.^42^ Additionally, others^52^ have reported the positive correlation that we observed between these metabolites. Indeed, xanthurenic acid is closely related structurally to kynurenic acid.^52^ Both metabolites are also ligands for the AHR.^50^ Importantly, the same rate-limiting enzyme, kynurenine aminotransferase produces kynurenic acid and xanthurenic acid from kynurenine and 3-hydroxykynurenine, respectively.^6,20^ Thus, our finding suggests that there is an equilibrium or cross-regulation of the steps leading to the production of kynurenic acid and xanthurenic acid.

We also observed 3 well-known relationships. The first is the link between the precursor, tryptophan and the host-derived kynurenine.^4,6,7,25,53^ In addition, the temporal reproducibility of their relationship is in agreement with reports of good to fair temporal reproducibility of tryptophan^42,43,54^ and kynurenine.^42,43^ In humans, the catabolism of tryptophan to kynurenine is through the action of the rate-limiting enzymes, tryptophan 2,3-dioxygenase in the liver, and indoleamine 2,3-dioxygenase.^1^ Moreover, this finding may reflect the maintenance of the tryptophan–kynurenine balance that is critical for physiological homeostasis.^55^ The second and third established associations, between kynurenine and 3-hydroxykynurenine, and between 3-hydroxykynurenine and 3-hydroxyanthranilic, represent the flux through the kynurenine pathway.^6,20^ It is quite interesting that this flux was stable over time in this study population. The human enzymes, kynurenine monooxygenase and kynureninase metabolize kynurenine to 3-hydroxykynurenine and 3-hydroxykynurenine to 3-hydroxyanthranilic acid, respectively.^6,20^ The temporal reproducibility of the relationship between these metabolites is in line with the reproducibility of kynurenine, 3-hydroxykynurenine, and 3-hydroxyanthranilic acid over 1 to 2 years follow-up in healthy postmenopausal women.^42^ Similar to our findings, previous reports have demonstrated the positive correlation between kynurenine and 3-hydroxykynurenine^4,7^ and between 3-hydroxyanthranilic acid and 3-hydroxykynurenine.^4,7^

Interestingly, all 5 relationships included 1 metabolite from the kynurenine pathway. This aligns with the well-acknowledged evidence that this pathway is the principal tryptophan metabolic pathway.^6,11^ Surprisingly, males and females did not share any temporally reproducible strongly connected metabolites across both network approaches. This sex-specificity corroborates sex-dependent tryptophan metabolism,^1^ and interaction of sex hormones with the kynurenine pathway.^25,26^ Thus, our finding is likely reflecting an appreciable underlying metabolic difference between the sexes that may be associated with the well-documented reports of sex-dependent differences in the gut microbiota composition, and gut microbial-microbial and microbial-host interactions.^56,57^ In addition, our finding of higher non-zero connection in the GGM of males as compared to females is in agreement with another study showing that the female network is less densely connected than the network in males.^58^ However, in the BN there were more probable connections in females than in males. This suggests that the density of connections between the sexes may depend on the network approach. Moreover, the adjustment of the networks for selected covariates did not materially affect the associations between the strongly related metabolites. This is in support of observations that extrinsic factors, such as diet or lifestyle have minimal impact on metabolite networks.^59^

Despite this study’s moderate sample size, there are indications that our findings are quite robust and reliable. First, there was no near-perfect collinearity between the metabolites as all correlation coefficients are below the implausibly high partial correlations of .8.^60^ Second, the strongest partial correlation between the metabolites was .59. This is supported by a report showing that the correlations of metabolomics data are usually less than .6 due to the systemic nature of metabolic control.^61^ Third, we observed 4.1% and 2.3% of all partial correlations ⩾ .3 in males and females, respectively, as compared to 1% reported by another study.^36^ Fourth, it is well documented that birth weight and length are correlated^62,63^ and energy and protein intake are closely related.^64,65^ Our study confirmed these relationships between birth weight and length, and between energy and protein intake. In fact, their association is strong, temporally reproducible, and consistent across sexes and network approaches. While it might seem that the different levels of assessment of covariates and metabolites drove the covariate–covariate and metabolite–metabolite associations and the stronger covariate–covariate associations; the fact that all variables were standardized suggests that these associations likely reflect factors that are part of similar natural processes. Finally, the fact that 3 of the 5 associations, which are across both sexes, have well-known relationships suggests that the novel relationships are unlikely to be spurious.

Considering the robustness of our findings, it is likely that these strongly related and temporally reproducible associations would also be observed in a larger study population with the same number of metabolites. In addition, urine and serum levels of tryptophan, kynurenic acid, and xanthurenic acid are positively correlated^66^; therefore, it is likely that the associations including these 3 metabolites would also be observed in the serum of this study population. Nonetheless, in future studies, the temporal reproducibility of the relationship between the metabolites profiled in the serum will be explored. In the future, we will attempt to unmask the underpinnings and determinants of these connections. We will also explore the links of these connections and their associated human and bacterial enzymes with health conditions. Moreover, our findings, particularly the 2 novel relationships generate important hypotheses that should be further investigated in independent larger human studies, animal models, and cell lines. It is also worthwhile that other studies confirm the network approach-specific relationships that we observed in this study. These are the relationships between kynurenic acid and quinolinic acid, between indole-3-acetamide and indole-3-acetic, and between indole-3-propionic acid and indole-3-carboxaldehyde.

There are several strengths of this study. First, it is an investigation exploring the temporal reproducibility of the relationship among tryptophan metabolites. To the best of our knowledge, no study until now has reported on the temporal reproducibility of the relationship among metabolites of tryptophan catabolism. Second, the age at baseline and follow-up were the same in the study population. This age homogeneity and the fact that our study population is young and apparently healthy suggests that age and chronic diseases are unlikely to confound our findings. Third, we adjusted the networks for important factors such as dietary intake and anthropometry that have been shown to influence tryptophan metabolism.^55^ Fourth, our rigorous statistical methodology of applying 2 network-based approaches facilitated obtaining reliable findings. Finally, the metabolites were profiled in the urine, a non-invasive biological sample that contains the final product of several metabolic processes. Nevertheless, there are some limitations of this study. The moderate sample size suggests that our findings should be interpreted with some caution. Moreover, the tryptophan metabolites that were quantified in this study are not comprehensive; as such, our findings may be biased. Although our definition of strong relationships between metabolites within the networks was with the use of literature-supported cutoffs, the use of different criteria might highlight different and additional relationships. Further, we included a few covariates to ensure that the covariate-adjusted networks do not fall too short of the recommended minimum of 3 participants per variable for a reliable exploration of networks.^67^ Despite the marginal attenuation of the association between the metabolites by our selected covariates, other covariates that we did not consider such as genetics might have considerable influence. Finally, our study population was limited to German adolescents from a higher socioeconomic status. This raises some concern on the generalizability of our results to other ethnicities, age groups, and socioeconomic status.

In conclusion, this epidemiological investigation underscores the capacity of network-based approaches to provide novel information on top of the known associations between tryptophan and its metabolites. These relationships, which include interaction of host and microbial metabolites, warrant evaluation in different physiological contexts. In addition, the metabolites in these relationships may represent a multi-biomarker panel that could be informative for health outcomes. Overall, our findings provide important progress in illuminating and understanding the interface between host and microbial tryptophan metabolism and warrant confirmation in different study populations.

## Supplemental Material

sj-docx-1-try-10.1177_11786469211041376 – Supplemental material for An Investigation into the Temporal Reproducibility of Tryptophan Metabolite Networks Among Healthy Adolescents Click here for additional data file. Supplemental material, sj-docx-1-try-10.1177_11786469211041376 for An Investigation into the Temporal Reproducibility of Tryptophan Metabolite Networks Among Healthy Adolescents by Kolade Oluwagbemigun, Andrea Anesi, Gerard Clarke, Matthias Schmid, Fulvio Mattivi and Ute Nöthlings in International Journal of Tryptophan Research

sj-docx-2-try-10.1177_11786469211041376 – Supplemental material for An Investigation into the Temporal Reproducibility of Tryptophan Metabolite Networks Among Healthy Adolescents Click here for additional data file. Supplemental material, sj-docx-2-try-10.1177_11786469211041376 for An Investigation into the Temporal Reproducibility of Tryptophan Metabolite Networks Among Healthy Adolescents by Kolade Oluwagbemigun, Andrea Anesi, Gerard Clarke, Matthias Schmid, Fulvio Mattivi and Ute Nöthlings in International Journal of Tryptophan Research

sj-png-1-try-10.1177_11786469211041376 – Supplemental material for An Investigation into the Temporal Reproducibility of Tryptophan Metabolite Networks Among Healthy Adolescents Click here for additional data file. Supplemental material, sj-png-1-try-10.1177_11786469211041376 for An Investigation into the Temporal Reproducibility of Tryptophan Metabolite Networks Among Healthy Adolescents by Kolade Oluwagbemigun, Andrea Anesi, Gerard Clarke, Matthias Schmid, Fulvio Mattivi and Ute Nöthlings in International Journal of Tryptophan Research

sj-png-2-try-10.1177_11786469211041376 – Supplemental material for An Investigation into the Temporal Reproducibility of Tryptophan Metabolite Networks Among Healthy Adolescents Click here for additional data file. Supplemental material, sj-png-2-try-10.1177_11786469211041376 for An Investigation into the Temporal Reproducibility of Tryptophan Metabolite Networks Among Healthy Adolescents by Kolade Oluwagbemigun, Andrea Anesi, Gerard Clarke, Matthias Schmid, Fulvio Mattivi and Ute Nöthlings in International Journal of Tryptophan Research
