# Supplementary figures and images for: An Investigation into the Temporal Reproducibility of Tryptophan Metabolite Networks Among Healthy Adolescents
Source: Int J Tryptophan Res. 2021 Sep 25;14:11786469211041376. doi: 10.1177/11786469211041376 (PMC8477685; doi:10.1177/11786469211041376)

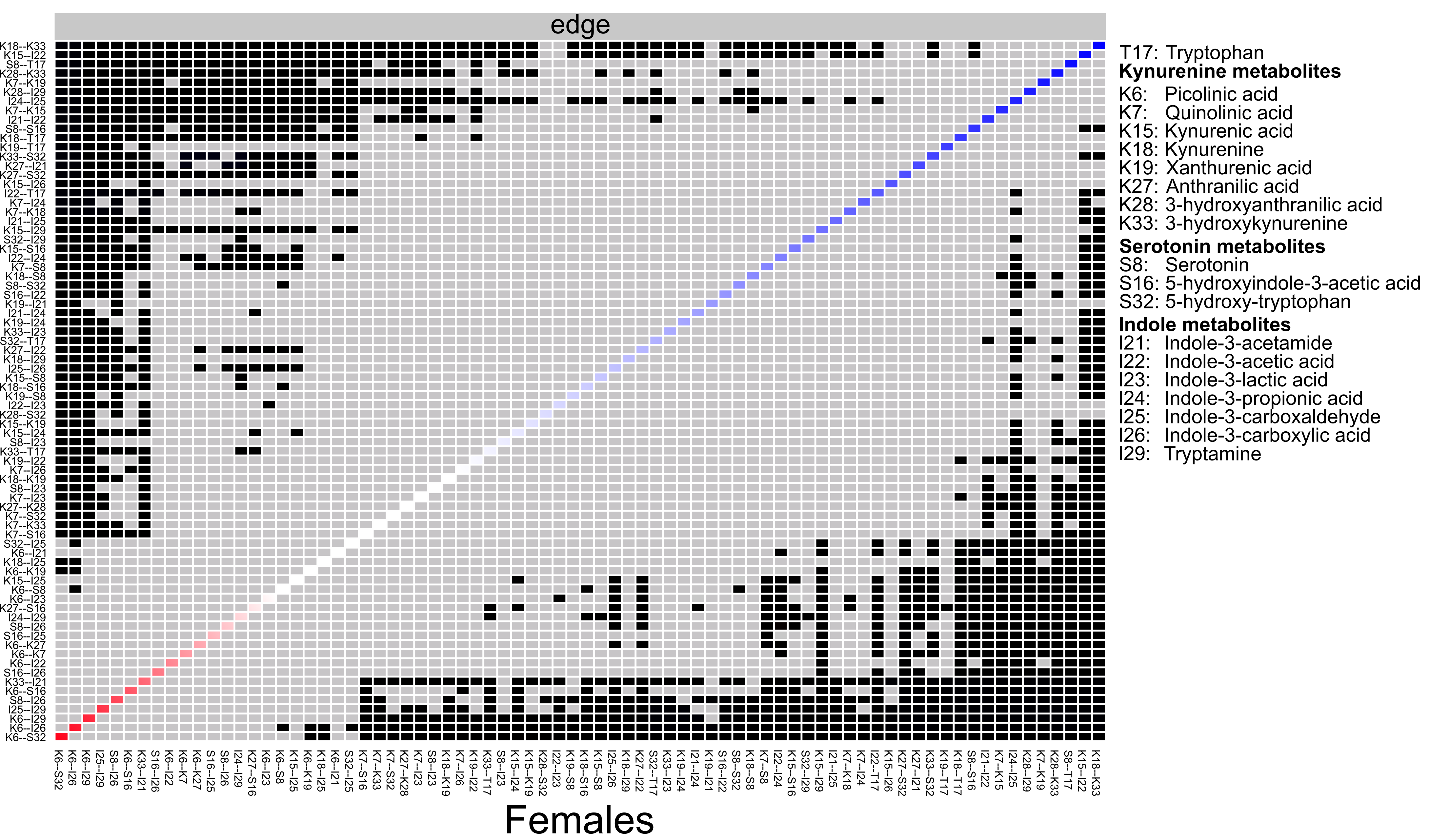

Supplement: sj-png-1-try-10.1177_11786469211041376 – Supplemental material for An Investigation into the Temporal Reproducibility of Tryptophan Metabolite Networks Among Healthy Adolescents [file sj-png-1-try-10.1177_11786469211041376.png]

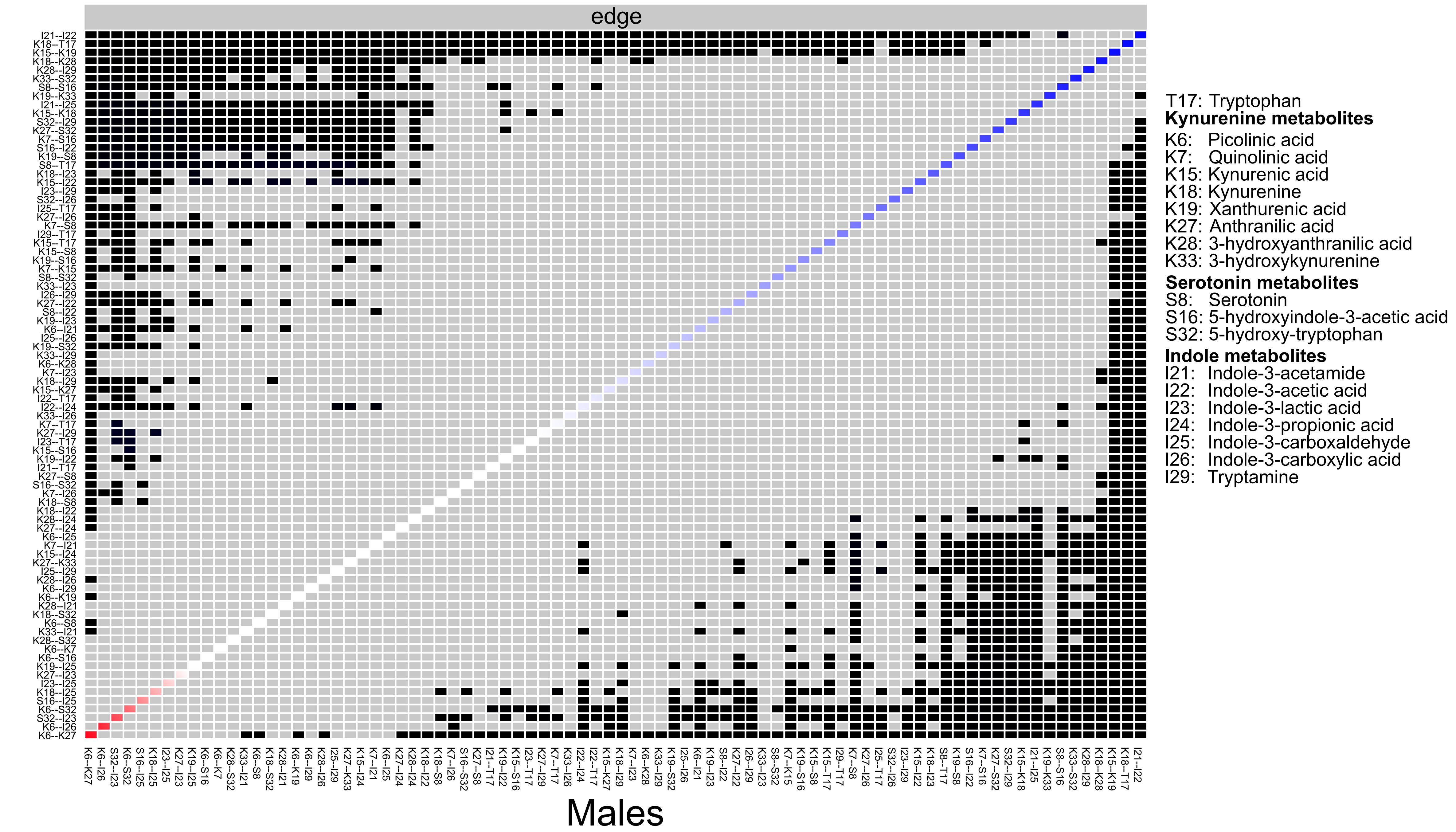

Supplement: sj-png-2-try-10.1177_11786469211041376 – Supplemental material for An Investigation into the Temporal Reproducibility of Tryptophan Metabolite Networks Among Healthy Adolescents [file sj-png-2-try-10.1177_11786469211041376.png]
