# Supplementary material for: An Investigation into the Temporal Reproducibility of Tryptophan Metabolite Networks Among Healthy Adolescents
Source: Int J Tryptophan Res. 2021 Sep 25;14:11786469211041376. doi: 10.1177/11786469211041376 (PMC8477685; doi:10.1177/11786469211041376)
Supplement: sj-docx-1-try-10.1177_11786469211041376 – Supplemental material for An Investigation into the Temporal Reproducibility of Tryptophan Metabolite Networks Among Healthy Adolescents [file sj-docx-1-try-10.1177_11786469211041376.docx]

25 May 2021

Dr. Dr. Kolade Oluwagbemigun

Nutritional Epidemiology

Department of Nutrition and Food Sciences

University of Bonn

Friedrich-Hirzebruch-Allee 7

53115 Bonn

Germany

Telephone: +49 (0) 228 73 69515

Email: koluwagb@uni-bonn.de

Dr. Gilles Guillemin,

Editor-in-Chief, International Journal of Tryptophan Research

Dear Dr. Guillemin,

Enclosed is an innovative and interesting epidemiological investigation entitled “An investigation into the temporal reproducibility of tryptophan metabolite networks among adolescents” for your consideration for publication in the International Journal of Tryptophan Research (IJTR).

The metabolites of tryptophan have dynamic interactions that may cut across multiple pathways. These interactions, especially those that involve host and microbial metabolites and that are strong and temporally reproducible could be of pathophysiological relevance. We believe that establishing such connections between metabolites of tryptophan would be an important research advance. By utilizing the two repeatedly collected 24-h urine among adolescents from the prospective DOrtmund Nutritional and Anthropometric Longitudinally Designed (DONALD) study, we quantified the concentration of tryptophan and 18 of its metabolites. We explored the interactions between the metabolites using two network-based approaches, the Gaussian graphical model and Bayesian network. Our data showed five temporally stable relationships. These include known relationships, between tryptophan and kynurenine and novel relationships, between kynurenic acid and indole-3-acetic acid depicting the interaction of host and microbial metabolites. Our findings provide important progress in illuminating and understanding the interface between host and microbial tryptophan metabolism and warrant confirmation in different study populations

We decided to submit this manuscript to the IJTR because focuses on tryptophan research and it has published several epidemiological investigations on tryptophan metabolism. This manuscript's content and results are submitted solely to the IJTR. They have not been published, in press, or submitted elsewhere and they have not been posted on the internet. All authors have no conflicts of interest to disclose. All authors have read and approved the final version of this manuscript.

Thank you for considering this manuscript. Please do not hesitate to contact us with any questions, comments, and suggestions. We look forward to hearing from you.

Yours sincerely,

Kolade Oluwagbemigun
